# The Impact of *Wolbachia* on Virus Infection in Mosquitoes

**DOI:** 10.3390/v7112903

**Published:** 2015-11-04

**Authors:** Karyn N. Johnson

**Affiliations:** School of Biological Sciences, The University of Queensland, Brisbane 4072, Australia; karynj@uq.edu.au; Tel.: +61-7-3365-1358; Fax: +61-7-3365-1655

**Keywords:** *Wolbachia*, *Drosophila*, mosquito, arbovirus, insect virus, symbiosis, antiviral protection, antiviral effects

## Abstract

Mosquito-borne viruses such as dengue, West Nile and chikungunya viruses cause significant morbidity and mortality in human populations. Since current methods are not sufficient to control disease occurrence, novel methods to control transmission of arboviruses would be beneficial. Recent studies have shown that virus infection and transmission in insects can be impeded by co-infection with the bacterium *Wolbachia pipientis*. *Wolbachia* is a maternally inherited endosymbiont that is commonly found in insects, including a number of mosquito vector species. In *Drosophila*, *Wolbachia* mediates antiviral protection against a broad range of RNA viruses. This discovery pointed to a potential strategy to interfere with mosquito transmission of arboviruses by artificially infecting mosquitoes with *Wolbachia*. This review outlines research on the prevalence of *Wolbachia* in mosquito vector species and the impact of antiviral effects in both naturally and artificially *Wolbachia*-infected mosquitoes.

## 1. Introduction

Understanding the factors that contribute to the transmission of arboviruses may facilitate strategies to limit the spread of disease. Mosquito transmission of viruses is impacted by interactions between the virus, host and other microbes. Presence of the endosymbiotic bacterium *Wolbachia pipientis* can interfere with microbial and parasite infection in insects, including viruses in mosquitoes (reviewed in [1,2,3,4]). As a result of this characteristic, there is increased interest in exploiting *Wolbachia* as a means of biological control of arthropod transmitted infectious pathogens (reviewed in [1,5,6]). This review is focused on the impact of both natural and artificial *Wolbachia* infection on the outcome of virus infection in vector mosquitoes.

## 2. *Wolbachia* in Insects

*Wolbachia* is an alphaproteobacterium predicted to infect more than 40% of insect species [7,8]. *Wolbachia* infection can have a wide range of impacts on insects [9]. An obligate intracellular bacterium, *Wolbachia* lives in the cytoplasm of host cells and is dependent on host cell resources for replication. The primary transmission route of *Wolbachia* is vertical inheritance through the cytoplasm of the maternal line, although horizontal transmission between insect species also contributes to *Wolbachia* prevalence [10,11,12,13,14]. Invasion of invertebrate populations is generally achieved via *Wolbachia* induced modification of host reproductive systems. Invasion into insect populations can occur very rapidly; for example, *Wolbachia* swept through Californian *Drosophila simulans* (*D. simulans*) populations in three years [15]. The ability to invade populations together with the recent finding that *Wolbachia* can interfere with virus transmission has led to interest in utilising *Wolbachia* to control mosquito transmission of arboviruses [1,3,6].

**Figure 1:**
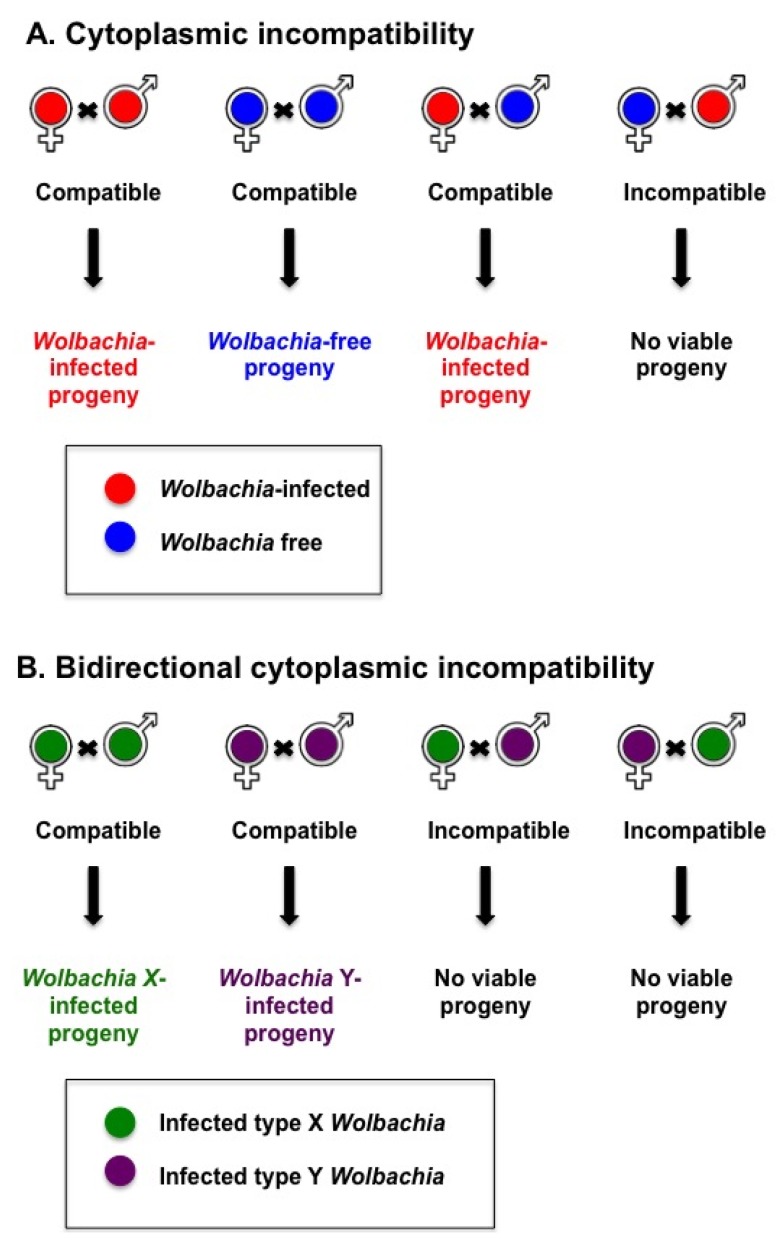
Cytoplasmic incompatibility induced by *Wolbachia* can lead to an increased number of *Wolbachia*-infected progeny in the population. (**A**) An incompatible cross arises when a male infected with *Wolbachia* mates with a *Wolbachia*-free female; (**B**) Crosses between parents infected with different *Wolbachia* strains will be incompatible when their *Wolbachia* strains are incompatible.

Cytoplasmic incompatibility (CI) is a prevalent *Wolbachia* reproductive manipulation in insects [16], which increases the proportion of *Wolbachia*-infected individuals in the population. *Wolbachia*-infected females can successfully mate with an uninfected male or male infected with the same or a compatible *Wolbachia* type (see Figure 1). CI occurs when a *Wolbachia*-infected male mates with a female that is either not infected with *Wolbachia* (unidirectional CI) or infected with an incompatible type of *Wolbachia* (bidirectional CI) [17]. That is, “if the male is infected with an infection (type) that is not present in his mate, it is an incompatible cross” [18]. In mosquitoes, *Wolbachia*-induced CI skews the population toward *Wolbachia*-infected females. In contrast to the female gametes, *Wolbachia* is not present in the male sperm. The molecular events that lead to CI are not completely clear but involve changes in condensation of male chromatin in *Wolbachia* free zygotes and lack of mitotic synchrony between the parental chromosomes [19,20,21,22]. In diploid insects such as mosquitoes, viable progeny are not produced from these eggs. CI is rescued in *Wolbachia*-infected eggs, as there is a restoration of synchrony between the male and female chromosomes, therefore producing diploid *Wolbachia*-infected progeny [20,22]. For biological control approaches, CI can be harnessed to establish *Wolbachia*-infected populations in the field [23].

## 3. *Wolbachia*-Mediated Antiviral Protection

*Wolbachia*-mediated antiviral protection was initially discovered in *Drosophila melanogaster* [24,25]. Flies infected with *Wolbachia* were protected from virus infection compared to paired groups of flies cured of their *Wolbachia* infection. In the presence of *Wolbachia* there is a significant delay in the mortality induced by the pathogenic RNA viruses Drosophila C virus (*Dicistroviridae*, DCV), Cricket paralysis virus (*Dicistroviridae*, CrPV) and Flock House virus (*Nodaviridae*, FHV) [24,25]. Accumulation of infectious DCV particles can be dramatically decreased early in infection, although a large change in FHV accumulation is not commonly observed. For the non-pathogenic Noravirus, a delay in virus accumulation was observed in the presence of *Wolbachia* without any impact on the survival of flies [24]. Thus presence of *Wolbachia* can have two different impacts on virus: firstly virus accumulation may be reduced/delayed, and secondly virus induced host mortality may be reduced/delayed. Both of these *Wolbachia*-induced effects are generally referred to as antiviral protection in *Drosophila*. However, the presence of some *Wolbachia* strains has no impact on virus-induced mortality or delay in accumulation [26]. In natural pairings, co-evolution of the host and *Wolbachia* mean that the contribution of each partner may be important for the outcome of antiviral protection.

In protective *Wolbachia*-host combinations, *Wolbachia* are found at high density. Different host-*Wolbachia* combinations have variable *Wolbachia* density in their insect hosts [27,28,29,30]. Protective *Wolbachia* are found at higher density than non-protective strains in *D. simulans* [26]; and if the *Wolbachia* density is experimentally decreased then protection is lost [27]. In *Drosophila*, *Wolbachia* density tends to be positively correlated with strength of antiviral protection and is sufficient to explain most of the variation in protection between *Wolbachia* strains [31,32]. In mosquitoes and mosquito cells *Wolbachia* density has also been implicated in antiviral effects [33,34,35,36]. The link between high *Wolbachia* density in the host and antiviral effects, leads to the hypothesis that all *Wolbachia* strains are capable of antiviral protection if a density threshold is reached, although this is yet to be tested experimentally.

The majority of studies assay *Wolbachia* density in whole insects; however, the importance of *Wolbachia* density to the outcome of infection may be at a cellular or tissue level. In addition to localisation in the germline tissues, *Wolbachia* has been identified in a number of somatic tissue types across insect species [27,28,30,36,37,38,39,40,41]. *Wolbachia* distribution is influenced by both host and *Wolbachia* factors (reviewed in [42]) and density can vary across tissues within a host [27]. There is some evidence to suggest that presence and density of *Wolbachia* in cells/tissues that are the site of arbovirus replication is important in determining the outcome of infection [35,36].

*Wolbachia*-mediated antiviral interference has been demonstrated for RNA but not DNA viruses. In *Drosophila*, *Wolbachia* had little impact on DNA virus infection, and the presence of *Wolbachia* enhanced a baculovirus-induced mortality in the African armyworm (*Spodoptera exempta*), a lepidopteran insect [24,43]. Apart from a decrease in virus-induced mortality, a range of other *Wolbachia*-mediated antiviral effects have been reported for RNA viruses in flies and mosquitoes [2,3] including reduced virus proliferation or transmission, reduced infection rate, no effect on virus infection or enhanced virus infection rates [24,25,26,31,32,34,36,41,44,45,46,47,48,49,50,51,52,53,54]. *Wolbachia*-mediated antiviral effects have been reported for viruses from a range of RNA virus families, including *Dicistroviridae*, *Nodaviridae*, *Flaviviridae*, *Togaviridae* and *Reoviridae*. Antiviral effects are commonly broad; that is, a host-*Wolbachia* combination effects one RNA virus is likely to affect other RNA viruses.

The potential mechanisms of *Wolbachia*-mediated antiviral effects are not completely clear and have been discussed in recent reviews [2,3]. The focus of this review is the incidence of antiviral effects in mosquitoes infected with *Wolbachia* either naturally or artificially, but a summary of the research of mechanisms is warranted. The diverse variety of viruses affected by *Wolbachia* infection of insects, suggests that the mechanisms are not likely to target interactions specific to one type of virus. It is also likely that the mechanisms are largely independent of *Wolbachia* strain given the importance of density and lack of phylogenetic congruency in strains that protect *Drosophila* [31]. The role of *Wolbachia* density in antiviral effects suggests there may be competition for resources between the virus, host and *Wolbachia* or remodelling of the host cell environment and there is some evidence for this competition [3,26,35,36,55]. Another potential mechanism for antiviral effects is immune stimulation by the presence of *Wolbachia*, in particular this has been implicated in mosquitoes transinfected with *Wolbachia* [36,49,56,57,58]. Interestingly, an increase in reactive oxygen species is observed in both mosquitoes and *Drosophila* infected by *Wolbachia*, and this is coincident with protection in *Drosophila* [58,59]. In addition, a role for microRNAs in controlling *Wolbachia*-mediated antiviral effects has been proposed [60,61]. Research on mechanisms of *Wolbachia*-mediated antiviral effects may be confounded by the difference between host species or mode of *Wolbachia* infection and further experimentation is required to delineate the important mechanisms.

## 4. Arboviruses in Mosquitoes

A subset of the over 3000 species of mosquitoes vector viruses that cause human disease (Table 1). The major mosquito-borne human pathogenic viruses come from three RNA virus families: *Flaviviridae* genus *Flavivirus*, *Togaviridae* genus *Alphavirus* and *Bunyaviridae* genera *Orthobunyavirus* and *Phlebovirus* (Table 1) [62]. These viruses are often found in both mosquitoes and animals, with virus replication occurring in both host types. Human disease occurs following transmission of the virus via a bite from an infected mosquito. For many of these viruses, humans are a dead-end host with viral population being necessarily maintained in other animal hosts. Viruses including dengue virus (DENV) and chikungunya virus (CHIKV) have adapted to a human-mosquito transmission cycle and no longer require amplification in other animals. While there are a complex range of factors that determine vector competence for transmission of arboviruses in mosquitoes [63], the presence of *Wolbachia* could influence vector competence by altering mosquito susceptibility to virus infection.

**Table 1:** Mosquito vectored arboviruses and their common vectors *.

Virus Family (Genome Nucleic Acid)	Genera	Examples of Arboviruses	Common Vectors
*Flaviviridae* (ss (+) RNA)	*Flavivirus*	Dengue virus	*Aedes aegypti*, *Aedes albopictus*
		Japanese encephalitis virus	*Culex* spp.
		St Louis encephalitis virus	*Culex* spp.
		West Nile virus	*Culex* spp.
		Yellow fever virus	*Aedes* spp.
*Togaviridae* (ss (+) RNA)	*Alphavirus*	Chikungunya virus	*Aedes albopictus*, *Aedes aegypti*
		O’nyong nyong virus	*Anopheles* spp.
		Semliki Forest virus	*Aedes* spp.
		Venezuelan equine encephalitis virus	*Aedes* spp., *Culex* spp.
*Bunyaviridae* (ss (−) RNA)	*Orthobunyavirus*	La Crosse virus	*Aedes triseriatus*
	*Phlebovirus*	Rift Valley fever virus	*Aedes* spp., *Culex* spp.

* For references see [62,64] and references there in.

## 5. The Distribution of *Wolbachia* in Vector Mosquitoes

*Wolbachia* was first discovered in the mosquito *Culex pipiens* [65] and is present in populations of various wild mosquito species. Surveys focused on disease transmitting mosquito genera identified *Wolbachia* in 7%–42% of the *Culex* pecies analysed and 0%–30% of the *Aedes* species analysed; until recently, no *Wolbachia* was detected in any of the tested *Anopheles* species [66,67,68]. It is interesting to note that *Wolbachia* is frequently detected in several of the common arbovirus vectors including the *Culex pipiens* complex and *Aedes* species including *Ae. albopictus* but not *Ae. aegypti*. The establishment of *Wolbachia* in some mosquito species can be impeded by the native microbiome and this may in part explain the absence of *Wolbachia* in some mosquito species in nature [69]. Improved methods of detection will likely lead to detection of *Wolbachia* in a wider variety of species. For example *Wolbachia* was recently detected for the first time in a limited number of *Anopheles gambiae* mosquitoes using high throughput sequencing of the 16s rRNA gene amplified from field caught mosquitoes [70]. The presence of *Wolbachia* in various arbovirus mosquito vectors raises the question of the impact of *Wolbachia* infection on arbovirus transmission in natural vector populations.

The ability to experimentally transfer *Wolbachia* into naïve hosts can create new vector-*Wolbachia* associations that do not occur in nature. This is attractive as a way of introducing *Wolbachia* into *Wolbachia*-free mosquitoes such as *Ae. aegypti*. In the laboratory *Wolbachia* can be transferred between insects by a process called transinfection (see [71]). *Wolbachia* is extracted from infected donor insects and injected into naïve insects. A stable transinfected insect line is established if the *Wolbachia* productively infects the female gonads and is passed from one generation to the next. Stable transinfection of *Wolbachia* into mosquito species is challenging, but has been successfully achieved for several species of mosquitoes including: *Ae. aegypti*, *Ae. albopictus*, *Ae. polynesiensis*, *Cx. pipiens*, and *An. stephensi* [41,50,72,73,74,75,76,77,78,79,80,81,82,83]. In addition, mosquitoes can be transiently transinfected by injection of *Wolbachia* into adult mosquitoes. In both cases *Wolbachia* invades various tissues of the mosquito and can be recognised as foreign, therefore stimulating the host immune responses [54,56,84].

The creation of a new stable association between a host and *Wolbachia* strain and can lead to phenotypic and genetic changes. In naturally infected insects, maternal inheritance of *Wolbachia* across many generations maintains a close association between the host and symbiont, leading to co-evolution and stable interactions. In comparison, theory predicts that new host-*Wolbachia* associations are likely to be maladapted [85], and artificial transfer of *Wolbachia* to a new host is known to induce novel host phenotypes [86,87,88], and can also result in a burst of changes in the *Wolbachia* genome [89]. However, adaptation in the new host can occur relatively rapidly [86,88].

## 6. The Intrinsic Effects of *Wolbachia* on Virus Infection in Mosquitoes

The presence of *Wolbachia* in mosquitoes has varied impacts on arbovirus infection. This is a complex tripartite system with contributions from the host, *Wolbachia* and virus on the outcome of virus infection. In addition, *Wolbachia* infections in the mosquitoes analysed in the laboratory can either be naturally occurring or introduced by transinfection. Thus studies of antiviral effects in mosquitoes may be confounded by the *Wolbachia* infection mode [2].

*Wolbachia*-mediated antiviral effects have been well documented in stably transinfected mosquitoes (Table 2). The mosquito species *Ae. albopictus*, *Ae. polynesiensis*, and *Ae. Aegypti* have been stably transfected with one or more of three *Wolbachia* strains (*w*Mel, *w*MelPop and *w*AlbB). These studies have included viruses from the families *Flaviviridae* (WNV, DENV and YFV) and *Togaviridae* (CHIKV) (Table 2). Arboviruses from the *Bunyaviridae* family are yet to be analysed in mosquitoes. A range of parameters can be examined for *Wolbachia* antiviral effects, these include: measuring the number of virus infected individuals, measuring virus load in whole or parts of mosquitoes, measuring dissemination and measuring virus in saliva as a proxy for transmission. In contrast to *Wolbachia*-mediated protection in *Drosophila*, since arboviruses have little impact on the survival of mosquitoes, protection against virus-induced mortality is not documented in mosquitoes. The impact of the presence of stably transinfected *Wolbachia* on the outcome of virus infection can range from a modest reduction in rate of infection amongst individuals or virus accumulation within infected individuals, to near complete interference with virus replication and transmission. Studies differ in the parameters tested and methods used, so it is difficult to make comprehensive comparisons across study systems. For example, in several cases antiviral effects were more prominent when virus was delivered orally rather than by injection [33,46]. However, in all cases where mosquito lines stably transinfected with *Wolbachia* have been analysed the presence of *Wolbachia* has decreased virus infection in at least one of the evaluated parameters [33,36,41,46,49,50,53,90].

Transient transinfection of *Wolbachia* into adult mosquitoes can lead to enhancement of virus infection. WNV infection rate was enhanced following transient transinfection of *w*AlbB into *Cx. tarsalis* mosquitoes [54]. Interestingly once the mosquitoes were infected with the virus, there was no impact of *Wolbachia* on virus accumulation, dissemination or transmission. This is the only enhancement of virus infection in mosquitoes that is linked to the presence of *Wolbachia*. It should be noted that transient transinfection is very different to natural stable infections where the host and *Wolbachia* have co-adapted to each other over many generations. It will be interesting to see if the enhancement in infection rate is limited to transiently transinfected mosquitoes. *Cx. tarsalis* mosquitoes are naturally *Wolbachia*-free [68] and stable transinfections of this mosquito have not been achieved so to date this comparison cannot be made. The possibility of arbovirus enhancement is an important consideration given there is one example of a natural *Wolbachia* infection stimulating increased susceptibility to a DNA virus in the African armyworm [43] and other examples of *Wolbachia*-induced enhancement of plasmodium infection in mosquitoes [84,91,92,93].

In contrast to stably transinfected mosquitoes, those naturally infected with *Wolbachia* do not ubiquitously exhibit antiviral effects (Table 2). *Ae. albopictus* mosquitoes naturally co-infected with *w*AlbA and *w*AlbB have similar total CHIKV or DENV loads to *Wolbachia*-free mosquitoes [34,51,52] and a small impact of *Wolbachia* on dissemination to the salivary glands was noted in one study for DENV [52]. *Wolbachia*-mediated antiviral effects for CHIKV were not stimulated by *w*AlbA and *w*AlbB introgressed into a new *Ae. albopictus* host background. The results of two studies on *Wolbachia* antiviral effects in naturally infected *Culex* mosquitoes were contrasting. While *w*Pip was shown to mediate reduced WNV loads and transmission in *Cx. quinquefasciatus* mosquitoes [94], no effects of natural *Wolbachia* infection were detected for *Cx. pipiens* mosquitoes infected with WNV [95]. Interestingly, the two studies were performed by the same research group and they identified that the laboratory population of *Cx. quinquefasciatus* used in the original study had much higher somatic density of *Wolbachia* than the recently caught *Cx. pipiens* mosquitoes used in the second study [95]. Further analysis of somatic *Wolbachia* density in recently caught *Cx. quinquefasciatus* mosquitoes was even lower than that of the *Cx. pipiens*. This suggests that while presence of *Wolbachia* in *Cx. quinquefasciatus* can lead to reduced vector competence, the *Wolbachia* density in natural *Culex* populations may not be high enough to support these antiviral effects. As a consequence, *Wolbachia* may not impact vector competence in the field. Taken together, these studies suggest that *Wolbachia* may not have a major impact on competence of mosquitoes with a naturally occurring *Wolbachia* infection to transmit arboviruses; however, a limitation is that there are few studies on recently caught populations of mosquitoes and further research in this area is required before conclusions can be made.

Comparison of transinfected and naturally infected mosquitoes may give insight into factors important for *Wolbachia*-mediated antiviral effects. Robust antiviral effects induced by *Wolbachia* strain *w*Mel transinfected into *Ae. albopictus* shows that this host can support *Wolbachia* antiviral effects [50,90]. However, natural *w*AlbA and *w*AlbB infection in this same host has either no or little impact on virus infection [34,51,52]. It is interesting that *w*AlbB transinfected into a different host, *Ae. aegypti*, is able to induce antiviral effects [49]. Thus, while both *Ae. albopictus* and *w*AlbB are individually competent partners for *Wolbachia*-mediated antiviral effects, no antiviral effects are demonstrated with this host-*Wolbachia* combination. This indicates that it is not a feature of either host or the *Wolbachia* strain *per se* which determines antiviral effects, but involves the interaction between the two. Transinfection of the *Wolbachia* into a new host in both these cases has stimulated antiviral effects. In mosquitoes there are two common effects of transinfection combinations that have antiviral effects: increased *Wolbachia* density and increased immune stimulation.

**Table 2:** Antiviral protection in mosquitoes naturally or artificially infected with *Wolbachia.*

Host Species	Mode of *Wolbachia* Infection	*Wolbachia* Strain	Virus *	Antiviral Effect **	Reference
*Culex quinquefasciatus*	Natural	*w*Pip	WNV	Reduced virus load and transmission	[94]
*Culex pipiens*	Natural	Not typed	WNV	No effect	[95]
*Culex tarsalis*	Transient transinfection	*w*AlbB	WNV	Enhanced infection rate	[54]
*Aedes albopictus*	Natural	*w*AlbA and *w*AlbB	DENV	No effect	[34]
	Natural	*w*AlbA and *w*AlbB	DENV	No effect on virus load, reduced dissemination	[52]
			CHIKV	No effect	[51]
	Introgressed	*w*AlbA and *w*AlbB	CHIKV	No effect	[90]
	Stable transinfection	*w*Mel	DENV	Reduced transmission	[50]
			CHIKV	Reduced transmission	[90]
*Aedes polynesiensis*	Stable transinfection	*w*AlbB	DENV	Decreased virus load, reduced transmission (compared to line naturally infected with *w*PolA)	[33]
*Aedes aegypti*	Stable transinfection	*w*MelPop	DENV	Reduced infection rate, virus load and transmission	[36]
			CHIKV	Reduced infection rate and virus load	[36]
			WNV	Reduced infection rate, viral load and transmission	[53]
			YFV	Reduced infection rate and virus load	[46]
		*w*Mel	DENV	Reduced virus load, dissemination and transmission	[41]
			CHIKV	Reduced virus load and transmission	[46]
			WNV	Delayed virus accumulation, reduced transmission	[53]
			YFV	Reduced virus load	[46]
		*w*AlbB	DENV	Reduced infection rate, virus load and transmission	[49]

* WNV, West Nile virus; DENV, dengue virus; CHIKV, Chikungunya virus; YFV, yellow fever virus. ** reduced transmission is measured by a reduction of virus load in the mosquito saliva; reduced infection rate indicates a decrease in number of individuals infected with virus, reduced virus load indicates that there is reduction in either viral genome copies or virus titre.

Immune stimulation has been noted following stable transinfection of mosquitoes, and has been proposed as a potential mechanism for antiviral effects [36,49,56,57,58]. *Wolbachia* infection also induces reactive oxygen species in transinfected *Ae. aegypti* [58] and naturally infected *Drosophila* [59]. An increase in reactive oxygen species corresponds with Toll pathway restriction of DENV infection leading to the suggestion that *Wolbachia* mediates anti-DENV effects through stimulation of the Toll pathway [58]. Contrasting this, broad immune stimulation is not observed in *Wolbachia*-mediated antiviral protection in *Drosophila*, either in naturally infected or heterologous transinfected flies [44,96,97]. In addition, no factors that influence *Wolbachia*-mediated protection have been identified by experimental disruption of *Drosophila* immune pathways including the Toll pathway [98,99,100]. Involvement of the Toll pathway in *Wolbachia*-mediated antiviral effects in transinfected mosquitoes could be directly tested through analysis of virus infection in Toll impaired mosquitoes.

Density is key to antiviral effects both in *Drosophila* and mosquitoes. The implication from several studies is that antiviral effects in transinfected mosquitoes are linked to an increase in *Wolbachia* density [39,90]. The principle of density being important for *Wolbachia*-antiviral effects is also well supported from both direct and indirect studies in *Drosophila* and cell culture systems [26,27,31,32,34,35]. It is not currently clear whether stable transinfection itself leads to an increase in density, or whether other factors such as a new host-*Wolbachia* association is involved.

Whether *Wolbachia*-mediated antiviral effects in mosquitoes will attenuate over time following transinfection remains to be determined [101]. Reduction in antiviral effects may eventuate if adaptation of *Wolbachia* to the transinfected host leads to a decrease in *Wolbachia* density or an attenuation of features that lead to the antiviral effects. Alternatively, the virus could evolve to “escape” the antiviral mechanisms mediated by *Wolbachia*. There is strong evolutionary pressure for the virus to overcome the *Wolbachia* antiviral effect and lack of evidence of strong effects in natural mosquito populations may suggest that evolutionary adaptation will lead to reduction of antiviral effects [101].

## 7. Conclusions

Currently, there is a contrast of antiviral effects reported in naturally versus artificially infected mosquitoes. The current literature suggests that natural infection of vector species with *Wolbachia* may not have widespread impact of arbovirus transmission. However, there are few studies on natural populations so it is not appropriate to draw strong conclusions at this point. In contrast, stable transinfection of *Wolbachia* into heterologous mosquito hosts clearly produces antiviral effects against arboviruses including DENV, WNV, YFV and CHIKV. These antiviral effects are likely related to increased *Wolbachia* density and possibly immune stimulation in the new host, although direct evidence for this is lacking. If antiviral effects are stimulated by the new *Wolbachia*-host association, it is possible that as adaption occurs these effects may decrease, which will be an important consideration for release of artificially infected mosquitoes as biocontrol for arbovirus transmission.
